# Supplementary material for: Are carbon emissions trading and green financial instruments synergistic? -Comprehensive quantitative research based on content analysis
Source: PLoS One. 2024 Mar 7;19(3):e0298601. doi: 10.1371/journal.pone.0298601 (PMC10919601; doi:10.1371/journal.pone.0298601)
Supplement: S1 Data — (ZIP) [file pone.0298601.s001.zip › Data/地级市Z_F工作报告.zip!/╣π╢1⁄2/╜╥╤⌠╩╨/└┤╫╘╣1⁄2╓┌║┼í╛┬φ┐╦╩2╛▌═°í┐_2004.docx]

各位代表：

　　我代表市人民政府向大会作政府工作报告，请予审议，并请政协各位委员和其他列席人员提出意见。

2003年工作回顾

　　2003年是极不寻常的一年，是我市经受考验、取得新成绩的一年。市政府在省委、省政府和市委的领导下，在市人民代表大会及其常委会和市政协的监督支持下，坚持以邓小平理论和“三个代表”重要思想为指导，全面贯彻中共十六大、十六届三中全会和胡锦涛总书记视察广东、张德江同志考察粤东重要讲话精神，围绕省委、省政府的战略部署和市第三次党代会提出的奋斗目标，按照“稳中求快，强化管理”的工作方针，一手抓防治非典 一手抓经济建设，加快实施工业立市、科教兴市、外向带动、可持续发展和区域协调发展五大战略，突出抓好十项重点工程和十项民心工程，取得了抗击非典和经济社会发展的双胜利。全市国民经济保持持续健康发展，全年生产总值488.55亿元，比上年(下同)增长10.8％；各项社会事业全面进步，基本完成了市三届人大一次会议提出的各项任务。

　　——工农业生产稳步发展，结构调整有效推进

　　农业和农村经济全面发展。全年农林牧渔业总产值52.8亿元，增长4.2％。农业生产条件继续改善，全年投入农田水利建设资金4.98亿元，恢复和改善灌溉面积6460公顷。种植结构进一步调整优化，果蔬、花卉、药材、水产品等农产品成为农村经济和农民收入新的增长点。农业标准化建设得到加强，制订了3个省级农业标准和6个市级农业标准，揭西京明有机茶生产基地获国家级认证，市农科所、玉湖华昌和揭西蓝天果蔬获无公害认证。新增蕉柑、柚木、蔬菜等７个市级以上标准化示范区。农业产业化步伐加快，28家年销售额千万元以上农产品加工企业，特别是15家省市级农业龙头企业的带动作用日益增强，85个农产品市场不断配套完善，农业社会化服务体系逐步建立健全。

　　工业发展速度加快。全年工业总产值896.25亿元，增长14%。工业用电量22.6亿千瓦时，增长43.3%。新增规模以上企业60家，总数达到480家。新增省级高新技术企业８家、省级民营科技企业15家，总数分别达到25家和90家。巨轮、运通公司通过上市公司资格评审，普宁市被国家定为纺织服装生产基地市。春南电器、康美中药饮片、榕泰化工新材料等一批技术改造和技术创新项目顺利推进。新增1个国家免检产品、4个省名牌产品，总数分别达到2个和9个；新增6件省著名商标、37件市知名商标，总数分别达到20件和119件。工业增加值占生产总值的比重达47.3%，工业主导作用更加明显。

　　——重点工程进展顺利，城乡建设取得新成绩

　　基础建设步伐加快。全年固定资产投资85.49亿元，增长8.7%。25个市级以上重点建设项目进展顺利，揭普高速公路、汕梅高速公路北新段建成通车，汕揭和潮揭高速公路前期工作顺利开展。榕江3000吨级航道整治工程通过省验收。汕揭、梅揭220千伏送电线路工程和西门、锡场、地都110千伏输变电工程竣工投入使用。榕江榕城围北堤、市区引榕干渠改造、惠来见龙海堤、凤山港海堤、横江水库、汤坑水库、石榴潭水库加固工程等项目建设取得成效。

　　城乡建设稳步推进。市岐山汽车站、黄岐山森林公园、榕江公园等项目建成或配套，市民盼望已久的市区污水处理厂和市区垃圾处理场等项目开工建设，市政设施进一步配套完善。“城中村”改造步伐加快。中心镇新一轮总体规划正在抓紧修编，建制镇近期建设规划有效推进，揭东炮台、普宁占陇下村、东山阳美村等样板村镇试点工作进展顺利。

　　——市场开拓取得成效，对外经贸势头良好

　　国内市场有效拓展。积极组织企业参加广东省（武汉）经贸洽谈暨产品展销会、技术进步成果展示会和其他商品交易会，成功举办第三届普宁（国际）衬衣节暨纺织服装交易会、第二届中华阳美（国际）玉器节。市果菜批发市场配套工程、揭东县城中心市场、普宁市供销物流配送中心筹建工作取得新进展。消费结构出现新变化，住房、汽车、通讯产品、医疗保健和旅游休闲等新的消费热点增长较快。全年社会消费品零售总额166.9亿元，增长12.2%。

　　对外经贸快速增长。全年外贸出口总额6.84亿美元，增长54.5%。机电产品和服装、鞋类等传统产品出口势头良好，对香港、美国、日本等主要贸易伙伴的出口进一步增长。

　　招商引资工作扎实开展，认真组织企业参加日韩经贸交流会等招商活动，引导企业加强与世界500强企业的联系和协作。全年新签外商直接投资项目32个，合同利用外资金额1.24亿美元，实际利用外资1.42亿美元。

　　——各项改革不断深化，市场经济体制进一步完善

　　国有企业改革稳步推进。国有中小企业逐步放开，国有资产监管体制进一步完善。揭阳糖厂、氮肥厂、电力厂等国有企业改制步伐加快，市棉纺厂、电焊机厂等企业改制的遗留问题得到进一步解决。

　　农村改革不断深化。农村土地资本化改革稳步推进，第二轮土地承包后续工作得到加强。农村税费改革成效明显，农民人均负担减少68.1元，减幅达72%，全市共减轻农民负担3.2亿元。撤并乡镇12个，完成了省下达的任务。

　　其他各项改革继续深入。第二轮行政审批制度改革深入开展，共削减审批事项261项，减幅39.48%。投资体制改革继续深化，社会投资基本建设项目登记备案制度初步建立。公共财政体制改革步伐加快，依法治税理财工作得到加强。产权、技术、劳动力、房地产等要素市场继续发展，有形建筑市场运作和管理逐步规范，工程招投标机制进一步完善。粮食流通等体制改革取得新进展。

　　——财税金融平稳运作，人民生活水平稳步提高

　　财政金融稳步增长。全市地方财政一般预算收入10.58亿元，增长8.38%；地方财政一般预算支出30.95亿元，增长20.87%。加上税收返还和上级补助，收支基本保持平衡。银企合作得到加强，中小企业贷款环境进一步改善。全市金融机构各项存款余额343.83%亿元，贷款余额174.88亿元，分别比年初增长16.74%和12.33%。

人民生活进一步改善。城乡居民储蓄存款余额282.33亿元，增长17.03%；城镇居民人均可支配收入7778元，增长11.8%；农村居民人均纯收入3834元，增长3.3%。十项民心工程扎实开展，社会保险覆盖面不断扩大，社会保障体系进一步完善。城镇登记失业率2.7%，比全省平均水平低0.2个百分点。农村困难家庭子女免收义务教育阶段书杂费政策得到落实，360名贫困生免费入读技工学校，228名贫困生就读高等院校扶贫班。农村群众饮水难等突出问题逐步得到解决。

　　——抗击非典取得重大胜利，社会各项事业全面进步

　　抗击非典取得显著成果。面对突如其来的非典疫情，我们沉着应对，果断决策，采取了一系列切实有效的防制措施，严防死守，科学防治。建立了突发公共卫生应急机制，完善了疫情信息报告网络，筹建了市公共卫生应急处理指挥中心，加强了疾病预防控制工作。广泛开展防治非典知识普及运动、全民爱国卫生运动和全民健身运动，有效地防止了疫情在我市的发生，使我市成为非典零病例和零疑似病例地区。医疗卫生事业得到了新发展。

　　社会各项事业协调推进。坚持以人为本，深入开展群众性精神文明创建活动，公民文明素质不断提高。成功举办第五届国际潮学研讨会，协助办好澳门潮汕美食节，进一步弘扬潮汕优秀传统文化。“普九”成果得到巩固提高，素质教育得到加强。改造薄弱学校工作基本完成，以县为主的教育管理体制改革稳步推进。科技事业有了新发展，列入国家和省各类科技计划40项，代理专利申请295项。“中国揭阳”政府门户网站荣膺“全国优秀社会公众服务网站”。榕城区新兴、榕华街道办事处被命名为省城市体育先进社区。国防动员、民兵预备役和拥军优属工作进一步加强，军政军民更加团结。统计、审计工作得到加强。文艺创作、新闻出版、广播电视、外事侨务、民族宗教、文史档案、气象地震、人民防空、优抚安置、赈灾救济等工作取得新进步。

　　——社会管理力度加大，可持续发展协调推进

　　“两大整治”深入开展。市场经济秩序进一步规范，打假、反走私、打击传销和变相传销等专项整治扎实开展，安全生产责任制得到进一步落实。社会治安综合治理力度加大，以打黑除恶、打击“两抢两盗”等为重点的专项斗争深入开展，“禁毒03工程”稳步推进，治安状况不断改善，我市成为全省刑事发案率较低的地区之一。信用体系建设得到加强，投资软环境继续改善。

　　生态管理不断加强。人口与计生工作稳步走上依法管理的轨道，全年人口出生率15.4‰，自然增长率10.5‰，实现省下达的人口与计划生育工作目标。榕江、练江、枫江等重点流域水污染治理工作得到重视，环境保护目标任期责任制进一步落实。森林覆盖率达51.5%，林业建设得到加强。土地资源治理整顿和保护力度不断加大，殡葬改革成果得到巩固，火化率达100%。

　　——民主法制建设稳步推进，政府自身建设进一步加强

　　民主法制建设继续推进。坚持向人民代表大会及其常委会报告工作、向人民政协通报情况，自觉接受监督。加强与工会、共青团和妇联等群众团体的联系，广泛听取各方面对政府工作的意见，科学化、民主化决策机制不断完善。及时有效办理省、市人大代表议案、建议和政协委员提案及建议意见149件。村（居）民自治逐步完善，政务公开、村务公开和厂务公开继续推进。“四五”普法宣传教育深入开展，法制工作得到加强。行政执法行为进一步规范，依法行政水平不断提高。

　　政府自身建设得到加强。廉政建设和反腐败斗争深入开展，廉政建设责任制进一步落实，一批违法违纪案件受到严肃查处。人民群众来信来访得到重视，一些群众反映强烈的热点难点问题得到有效解决。机关作风建设和效能建设稳步推进，公务员管理逐步规范，服务水平不断提高，廉洁勤政、务实高效的政府形象进一步形成。

　　各位代表！去年，我们经受了多方面的严峻考验，取得了抗击非典的重大胜利，保持了经济社会的持续发展，特别是群众广为关注的一些重大项目，如惠来电厂、市区垃圾处理场、市区污水处理厂等取得了重大突破。这些成绩的取得，是省委、省政府和中共揭阳市委正确领导的结果，是各级人民代表大会及其常务委员会和人民政协监督支持的结果，是全市人民团结一心、攻坚克难、奋力拼搏的结果。在此，我代表市人民政府向全市人民，向各民主党派、人民团体、工商联和各界人士，向中央和省驻揭单位，向驻揭人民解放军、武警官兵、人民警察表示崇高的敬意！向长期关心支持揭阳现代化建设的港澳台同胞、海外侨胞、海内外乡亲和友好人士表示衷心的感谢！

　　回顾一年的工作，我们清醒地认识到，我市经济社会发展中还存在不少困难和问题。主要是：经济运行的质量和效益不高，产业竞争力较弱，企业规模偏小；县域经济实力不强，发展不平衡；财政收支压力较大，镇村集体经济薄弱；就业和社会保障任务较重，城乡部分群众的生产生活仍然困难；城市建设、生态环境、社会治安有待继续加强，总体投资环境仍不理想；政府职能转变相对滞后，机关作风建设需要进一步加强，等等。对这些问题，我们必须高度重视，认真采取措施加以解决。

2004年工作安排

　　2004年是实施“十五”计划的关键一年，也是我市打基础、抓项目、促发展、树形象的重要一年。着眼当前，放眼未来，我们有着难得的发展机遇。世界经济结构调整步伐加快，我国市场经济快速发展，内地与港澳更紧密经贸合作全面实施，“大珠三角”和“泛珠三角”区域协作日趋紧密，省委、省政府对东西两翼扶持力度加大，周边地区竞相发展，全市人民致富奔康的愿望更加强烈。形势逼人，催人奋进。我们必须科学判断形势，正确把握发展态势，抓住加快发展的机遇期，进一步增强抢抓机遇、加快发展的紧迫感和责任感，自觉克服害怕困难、萎靡不振、安于现状、观望等待、无所作为等各种消极思想倾向，立大志，想大事，创大业。敢于迎难而上，主动迎接挑战，积极化解矛盾。坚持在解放思想中统一思想，坚持用发展的办法解决前进中的困难和问题，坚持与时俱进，以奋发有为的精神状态，真抓实干，创造性地开展工作。

　　今年市政府工作的指导思想是：以邓小平理论和“三个代表”重要思想为指导，认真树立和落实科学发展观，全面贯彻中共十六大、十六届三中全会、省委九届四次全会和张德江同志考察粤东重要讲话精神，按照市第三次党代会和市委三届二次全会提出的发展战略和目标任务，以抢抓机遇、加快发展为主题，以加大固定资产投资为突破口，以工业发展为龙头，培植壮大县域经济，提高经济综合实力和竞争力。正确处理好改革发展稳定的关系，统筹发展各项社会事业，不断提高人民生活水平，切实加强精神文明建设和民主法制建设，营造良好发展环境，努力促进全市经济社会全面、协调和可持续发展。

　　今年我市经济社会发展预期目标为：全市生产总值增长10%；地方财政一般预算收入增长7%；社会消费品零售总额增长11%；固定资产投资增长13.6%；外贸出口增长15%；城镇登记失业率控制在3%以内；人口自然增长率控制在8‰以下；城镇居民人均可支配收入增长10%；农村居民人均纯收入增长4%。

　　贯彻上述指导思想，实现全年预期目标，必须围绕市委的工作部署，努力做到发展有大思路、大投入、大项目、大环境、大热点，力促工业发展、城区建设、文化教育实现新突破，突出抓好关系全局的市区防洪工程、惠来电厂工程、潮汕民用机场工程、两个环保工程、文化设施工程、教育卫生工程、市区道路改造工程、文明建设工程、交通管理工程、固本强基工程等十件实事，以实实在在的工作成效取信于民。为此，要切实做好十方面工作：

　　一、加大固定资产投入，形成大发展拉动力

　　积极筹集建设资金。抓住国家继续实施积极财政政策的机遇，创新投融资机制，推动国债资金、银行资金、财政资金和民资、外资“五个轮子”一起转，鼓励社会资本积极参与基础设施和社会文化设施的建设和经营，精心组织好项目的推介和招商工作，多渠道、多形式筹措建设资金。以突出抓好十件实事为主线，全面推进重点项目建设。着力加强水利、交通、能源等基础设施，力争今年新增100个投资1000万元以上项目，固定资产投资总额100亿元以上。

　　以市区防洪工程建设为龙头，加大水利设施投入。按照堤路城综合开发的思路，总体规划，分项设计，重点突破，整体推进。全民动员，全力以赴推进市区防洪工程建设，力争五年内完成预期目标。今年要着力推进榕城围北堤和南堤、东山围、渔湖围北堤和南堤等市区首期防洪工程项目建设，加快炮台海堤、金凤联围、蜈蚣岭水库、磐岭围、棉湖联围等水利项目建设进度，提高城乡水利防灾减灾能力。

　　着力加大交通设施投入，完善交通网络。争取潮汕民用机场列入省近期建设重点项目，成立协调机构，确定投资主体，尽快开展前期工作。认真协助抓好潮揭、汕揭高速公路建设，抓好国道206线揭丰段、国道324线沙池段、省道236线池揭段改造建设，加快京棉公路和南河大桥加宽工程建设进程。积极规划、拓宽改造揭陆线、池安线。加快村镇公路建设步伐，确保通行政村硬底化公路按期完成。

　　以惠来电厂为突破口，大力推进能源项目建设。惠来电厂装机总容量10×60万千瓦，首期建设4台机组，总投资115亿元，是资本密集型投资项目，对全市经济社会发展具有巨大的拉动力和影响力。今年要完成评估、申报、地表清理、工程设计等工作，力争尽快获得国家立项批准建设。争取惠来10万千瓦特许权风电场开工，跟踪落实好省核电站选址落户我市工作。做好揭阳500千伏、普宁龙秋和市区东泮220千伏等输变电工程前期工作，加快军埠、磐东、东方等8项110千伏输变电工程建设进度，开工建设云路、华湖等4项220千伏和里湖、群光等7项110千伏输变电工程。加快市区电网建设和第三期农网改造。

　　二、大力发展民营工业，着力提高工业化水平

　　以骨干企业形成功能区。进一步落实加快民营经济发展的政策措施，营造良好的政务、法制、市场、治安和舆论环境。抓好民营企业30强评选活动，发挥示范带动作用。鼓励民营企业做强做大，引导骨干企业推进科技、体制和管理创新，扩大规模、强健主业和形成核心竞争力。加强对现有上市公司的服务和扶持，鼓励、支持具备条件的龙头骨干企业改制上市。全力推进化工、汽车、轮胎、玉器、鞋材和电子等工业城的建设，形成专业生产功能区，提高企业的规模化和组织化程度。

　　以支柱产业促进工业结构优化升级。大力推动企业技术改造和技术创新，进一步巩固和提升传统产业特别是特色产业的发展水平，积极培植发展电子信息、生物制药、新材料、光机电一体化等新兴产业。创造条件，积极引进重化项目。全力推进纺织服装、五金不锈钢、模具生产、塑料制品、医药生产和食品饮料等生产基地的建设，提高产业竞争力。强化名牌带动，做大现有品牌，积极引导有条件的企业申报知名商标、著名商标、驰名商标以及国家、省名牌产品和国家免检产品，培育形成一批品牌产品。力争今年新增100家规模以上企业，新增100个投资500万元以上工业项目。

　　以园区建设推动产业集群发展。进一步完善园区基础建设和配套建设，调整园区布局，整合有效资源。落实各项优惠措施，加强规范管理，提升园区的档次和品位。全力推进揭阳经济开发试验区、揭东经济开发试验区、揭阳（惠来）沿海经济开发试验区等工业园区建设，增强园区集聚能力，以园区吸引项目，以项目支撑园区发展，形成产业集群，努力在产业规模、配套能力、核心技术、品牌战略、市场份额等方面取得新的突破。

　　三、统筹城乡协调发展，全面繁荣农村经济

　　做大特色产业，发展壮大县域经济。加大指导和协调力度，充分挖掘和整合县域的区位优势和资源优势，因地制宜，构建各具特色的产业、产品和品牌，培育和壮大县域经济。发挥普宁轻工业基础较好、商贸流通活跃的优势，力争把普宁建成轻工业生产基地和流通基地。做大揭东开发区品牌和特色农业规模，力争把揭东建成区域性的新兴制造业基地和“三高”农业基地。大力开发临海资源，推动海洋经济发展，把82公里海岸线规划好、控制好、开发好，力争把惠来建成海洋经济基地和能源基地。积极推进山区资源开发利用，力争把揭西建成生态工业、生态旅游和生态农业基地。做活“侨”字文章，壮大普侨区和大南山侨区经济实力。

　　调整农业结构，提高产业化水平。以最严格的措施管好、用好耕地，依法加强基本农田保护，保障粮食生产安全，办好揭东炮台、揭阳经济开发试验区两个万亩片粮食高产示范区。继续调整农业生产结构，大力发展6大农业支柱产业和果蔬、花卉、茶叶、水产等特色产业，拓展品牌农业。推进农业规模化和标准化生产，抓好普宁中药材种植、惠来海水养殖、揭西罗非鱼养殖和农业商品基地、农业标准化示范区建设。以培育扶持15家省市级农业龙头企业为重点，构建农产品加工体系。搞好农产品市场建设，发展市场中介组织，建立健全农业信息网络，完善农业社会化服务和支持体系。

　　增加农民收入，减轻农民负担。按照“工业进园、住宅进区、农民进厂”的思路，加快发展包括县城镇在内的16个中心镇和专业镇，推进农村城镇化进程。拓宽农民就业渠道，推动农业人口向城镇转移，农村劳动力向工业和园区集中，增加农民非农收入。抓好农村富余劳动力技能培训工作，力争今年转移就业4.2万人。严格执行“五取消、一改革、一种税”，巩固农村税费改革成果。继续整顿清理涉农行政性收费和经营服务性项目，完善涉农收费公示制。继续抓好农村中小学乱收费和对农民建房乱收费等专项治理，防止农民负担反弹。继续落实有关扶贫政策措施，加大扶贫开发力度。

　　四、加大城区建设力度，提升城市档次和品位

　　加强城市规划管理。抓紧修编好市区总体规划，加强市区181平方公里规划控制区、58平方公里规划建成区修编、衔接工作。加快实施《“两河四岸”控制性详细规划》、《揭阳市中心城区近期建设专项规划》，抓紧编制市区排水排污、道路交通、绿化等专项规划，制订好功能区规划和控制性详规。认真实施《揭阳市城市规划管理规定》和其他管理法规。以交通综合整治作为城市管理的切入点，争取今年取得明显成效，并以此为契机，全面整治市区脏乱差问题，塑造城市新形象。

　　推进市政设施建设。树立经营城市理念，放开经营性项目建设市场，引导鼓励民资、外资采取多种形式参与市政建设，对公交线路、户外广告经营权和地下管道铺设权等资源实行“特许权经营”。加强市区土地控制工作，严厉查处非法用地行为，切实保护市区土地存量，提高城市土地资源的利用水平。围绕抓好十件实事，全力推进环境保护工程、市政道路美化工程和穿城公路改造工程建设。走市场化、社会化道路，采用BOT运作模式，引进民资和外资，积极争取国债资金和专项补助资金，多渠道筹资加快市区垃圾处理场、市区污水处理厂建设步伐，确保垃圾处理场第一期工程年中投入使用，争取明年完成污水处理厂厂区建设。下决心搞好市政道路建设，力促城市功能上档次、上水平。今年要突出抓好黄岐山大道、临江北路、仁义路等3条市政道路的美化工程，加快临江南路改造、莲花大道南段、进贤门大道东段、马牙路、晓翠路等主要干道的建设和绿化配套，推进凤潮文化休闲区和丹凤商贸购物区建设，尽快形成城市主体功能区，进一步提升城市品位。

　　加快市区经济发展。调整优化市区发展布局，增强市区经济实力和综合竞争力，发挥龙头带动作用。抓好市区工业园区建设，引导企业向工业园区集中。进一步发展和提升电子电器、五金模具、塑料制品、不锈钢等优势产业，做大做强市区工业。推进商贸设施建设，完善购物环境，使市区逐步成为周边地区的重要商贸物流中心。积极规划建设进贤门、步行街、学宫、城隍庙、黄岐山森林公园等市区旅游圈，大力发展市区旅游业。加快发展城郊型农业，增加市场供给。

　　五、千方百计招商引资，努力开拓国内外市场

　　大力招商引资。创新招商方式，探索开展企业化、市场化、社会化招商路子。层层落实招商引资责任制，实行招商引资主体下移，一级抓一级，把招商引资责任制分解落实到县、镇和各经济功能区。修订完善招商引资优惠办法，最大限度地为外商投资提供优惠条件，调动社会各方面招商引资积极性。在继续吸收港澳台投资的同时，积极引进欧美日等发达国家和地区的资金，力争世界500强企业项目落户我市实现零突破。引导外资参与国企改革，拓宽服务业吸收外资的渠道。努力创造条件，积极承接珠三角特别是广州、深圳、东莞等地区的产业转移。鼓励现有外商投资企业增资扩产。

　　努力扩大出口。主动适应出口退税新机制，推动外贸出口持续稳定协调发展。以发展加工贸易出口为重点，在稳定一般贸易出口的基础上，提高加工贸易比重，带动出口稳步增长。贯彻落实鼓励和扶持外贸出口政策措施，调整优化出口产品结构，培育更多的名、优、特出口产品。加强部门沟通协调，推进“大通关”建设，营造良好经营环境。

　　扩大消费需求。清理不利于消费的政策措施，改善消费环境。加快建设市服装产品检测站，健全产品质量监督机制。加强市场流通设施建设，巩固提高市水果批发、普宁纺织服装、中药材等专业市场，规划建设市区不锈钢、水产禽畜和惠来水产品等市场。大力发展旅游业，加快京明旅游度假区、黄满石祭瀑布旅游区、揭东明月湖旅游度假区等旅游项目建设步伐。高度重视现代物流业的发展，积极发展金融、证券、保险等服务业，推进连锁经营、物流配送、网上销售等现代流通业。培育和扩大住房、汽车、电信等消费热点，增强消费需求对经济发展的拉动力。

　　积极拓展国内市场。继续采取“走出去”与“请进来”相结合方式，积极参加省举办的各类经贸洽谈会、展销会、博览会，认真组织企业到广州、深圳、东莞等地举办推介会，继续办好中国（普宁）中药交易大会、普宁（国际）衬衣节暨纺织服装交易会、中华阳美（国际）玉器节，筹办揭西生态旅游文化节，大力发展会展经济。推进闽粤赣三省十三市区域经济协作。抢抓CEPA先机，加强同“泛珠三角”和“大珠三角”区域的联系和交流。引导企业参与东北地区等老工业基地振兴和西部大开发。

　　六、狠抓增收节支，确保财税金融稳健运行

　　努力增加财政收入。大力培育地方税源，提高财政收入占生产总值的比重。严格依法治税，加大增值税、营业税、企业所得税、个人所得税等税收征管力度，强化地方小税种征缴，规范非税收入管理，完善国有资产收益收缴管理制度。引进激励机制，调动地方发展经济和组织收入的积极性。大力清理经济户口，确保应收尽收。

　　强化财政支出管理。依法编制财政预算，严格预算管理，按照“保重点、压一般”的原则，调整支出结构，确保人员工资、社会保障等重点支出资金需要，一般性支出今年实行零增长。积极推行和完善政府采购制度，规范采购行为。进一步健全公共财政体制，推进财政管理制度改革和创新。规范财经秩序，加强审计监督，严肃财经纪律。

　　加强金融工作。继续做好地方金融风险防范和化解工作，加大清收欠款和资产变现力度，妥善处置地方金融不良资产和政府债务。加强与金融部门的协调和沟通，争取更多的银信资金支持地方经济社会发展。进一步加强金融监管，稳妥推进金融体制改革。

　　七、深化体制改革，为经济社会发展注入新动力

　　深化国有企业改革。以产权制度改革为突破口，逐步建立归属清晰、权责明确、保护严格、流转顺畅的现代产权制度。积极引导民资、外资参与国有企业股份制改造，促进国有企业投资主体多元化，大力发展混合所有制经济。继续做好揭阳糖厂、氮肥厂、电力厂、饲料厂、石板材厂等国有企业改制工作，妥善解决好棉纺厂、电焊机厂改制的遗留问题。积极探索国有资产监督管理的有效形式，建立健全国有资产科学管理体制。推进垄断行业改革。

　　深化行政管理体制改革。按照市场机制的要求，进一步规范事权、财权和人事权。加快政企分开，进一步把不该由政府管的事交给企业、社会组织和中介机构，更大程度地发挥市场在资源配置中的基础性作用。继续深化行政审批制度改革，加快发展电子政务，创新行政管理手段。稳步推进市县政府机构改革，全面推进城市社区建设。

　　深化市场体系改革。严厉打击传销和变相传销、制假售假、走私贩私、偷税逃税、侵犯知识产权等违法犯罪活动，进一步规范市场主体和市场行为。促进以道德为支撑、以产权为基础、以法规为保障的社会信用体系建设。大力发展资本、产权、人才、土地、劳动力、有形建筑等要素市场，加快培育发展各类社会中介组织和行业协会，健全社会服务体系。

　　深化农村改革。完善农村土地征用制度，稳妥推进土地资本化改革，进一步落实土地承包经营权。积极探索农村土地股份合作和土地合理、有序流转的有效形式，推进农业适度规模经营。妥善解决乡镇教育欠债、镇村其他负债等问题。

　　八、坚持以人为本，推进社会事业发展

　　切实加强精神文明建设。加强理想信念教育，坚持用邓小平理论和“三个代表”重要思想武装头脑、指导实践、推动工作。弘扬“新时期广东人精神”和“聚三江人心，创时代伟业”的揭阳精神。加强以诚信为重点的公民道德建设，广泛宣传《揭阳市市民文明公约》、《揭阳市公民道德规范》，不断提高公民的文明和道德素质。扎实开展文明城市、文明村镇和文明行业等群众性精神文明创建活动，引导城乡居民形成科学、健康、文明的生活方式。

　　大力发展文化教育。围绕抓好十件实事，全力推进文化体育工程和教育卫生工程建设，抓紧筹建市体育馆、广播电视中心、图书馆等文化体育设施和揭阳一中新校区、市卫生学校新校区、市人民医院住院大楼、市职业技校等教育卫生设施。市体育馆今年要完成征地、设计等前期工作，力争用2年时间建成。市广播电视中心今年要完成选址、征地、设计等前期工作，力争用3年时间建成，作为建市15周年的献礼工程。揭阳一中新校区今年要动工建设，力争２年内建成，2006年春季开学投入使用。改革文化管理体制，落实支持公益性文化事业、文化产业发展的政策措施，大力推进传媒、音像、出版、咨询、会展、旅游等特色优势文化的产业化进程，使之成为我市新的经济增长点。加强基层文化建设，加大文物保护力度，推动对外文化交流。巩固提高“普九”成果，重视薄弱学校改造，全面推进素质教育。制订和实施全市普通教育规划，着力加快高中阶段教育发展步伐。努力办好揭阳职业技术学院。积极发展继续教育、远程教育和网络教育。引导、鼓励和规范社会力量办学，大力发展民办教育。加大人才资源的开发、培养和引进力度，健全各类人才的选拔任用、考核评价、激励监督机制，吸引更多人才来揭工作和创业，为经济社会发展提供智力支持。

　　统筹发展各项社会事业。加大科技攻关和成果转化力度，完善以企业为主体的科技创新体系。加强科普知识宣传工作，依法保护知识产权。完善公共卫生体系，推进疾病预防控制体系、疫情信息网络体系、卫生执法监督体系和医疗救治体系建设，健全突发公共卫生事件应急处理机制。抓好全民安康工程，突出做好非典等各种传染病的防治工作，严防禽流感感染到人。继续深化医疗卫生体制改革，深入开展全民爱国卫生运动。大力改造薄弱乡镇卫生院，建立和完善新型农村合作医疗制度，提高农村合作医疗的人口覆盖率和保障水平。广泛开展全民健身活动，大力发展竞技体育，促进体育事业和体育产业发展。加强老龄、妇幼和残疾人事业，维护妇女儿童的合法权益。加强全民国防教育和防空建设，做好拥军优属工作。高度重视做好外事、侨务、台务、民族、宗教、统计、民政、档案、方志、文史等工作。

　　九、高度关注民生民计，努力保持社会稳定

　　关心群众生活。全面落实促进就业和再就业各项优惠政策措施。加大就业岗位开发力度，力争全年净增就业岗位2万个，再就业率60%以上。进一步扩大社会保险覆盖面，实施企业养老、失业保险市级统筹，强化社保基金征缴和监管，确保下岗职工基本生活费和离退休人员养老金按时足额发放，进一步完善优抚保障制度和城乡最低生活保障制度，做好帮扶弱势群体工作，推进城乡社会福利机构建设，大力发展济困助残事业。规范拆迁程序，切实保护群众合法权益。扎实推进市第二水厂供引水工程、揭东三镇供水管网、普宁改水工程、揭西棉湖供水工程和惠来岐石改水工程建设，切实解决群众饮水难、入学难、就医难等问题，关心和安排好困难群众的生产生活，确保全市人民无饥寒。

　　优化发展环境。加强环境保护，强化榕江、练江、龙江等流域的水源保护和综合治理，重点保护好饮用水源，提高水环境质量。加大环境保护执法力度，继续整顿和清理小电镀、小漂染等污染企业，巩固和提高“一控双达标”成果。加强林业建设，提高森林覆盖率。建立和完善自然灾害监测预警机制，合理开发利用土地、森林、矿产、海洋等资源，坚决杜绝乱开乱采、破坏资源的行为。抓好人口与计划生育“一法三规”和《条例》的贯彻落实，进一步建立健全 “依法管理、村民自治、优质服务、政策推动、综合治理”的工作机制，强化计生基层组织建设，推动工作重心下移，力争把人口出生率控制在13.5‰以下。继续抓好殡葬改革和配套设施建设，巩固殡葬改革成果。

　　维护社会稳定。做好人民群众来信来访工作，正确处理人民内部矛盾。建立社会纠纷动态监管机制，及时调处民事纠纷。严密防范、坚决打击“法轮功”邪教组织破坏活动。坚持重典治乱，继续保持严打高压态势，深入开展打击“两抢两盗”、“涉黑涉恶”、利用“六合彩”赌博等专项斗争，继续实施“禁毒03工程”，健全治安防控体系。切实抓好安全生产，落实安全生产责任制，增强交通和消防安全意识，坚决消除安全生产隐患，确保人民生命财产安全和社会稳定。

　　十、加强民主法制建设，提高依法行政水平

　　推进民主制度建设。自觉接受人大及其常委会的监督，认真落实人大及其常委会的决议、决定，及时向人大及其常委会报告工作。积极支持人民政协履行政治协商、民主监督、参政议政职能，主动听取各民主党派、工商联、无党派人士及各人民团体的意见建议。切实办好人大代表议案、建议和政协提案。逐步建立重大事项社会公示制度和社会听证制度，扩大公民有序的政治参与，积极推进政务公开、村务公开和厂务公开，拓宽反映社情民意的渠道。完善村民居民自治，健全企事业单位民主管理制度，确保人民群众依法参与民主选举、民主决策、民主管理、民主监督，维护人民群众当家作主权利，促进政治文明建设。

　　严格依法行政。认真抓好以宪法为核心的法制宣传教育，贯彻实施行政许可法，规范政府行政行为。清理现行有关行政许可的规定和行政许可实施机构，修改或废止与行政许可法不一致的规定。规范、完善相对集中行政处罚权制度，强化对行政许可的监督。进一步加强政府法制工作和执法监督，规范行政复议和行政应诉，提高依法行政水平。

　　加强政府自身建设。理顺和转变政府职能，加强政府对公共事务的管理，提高政府应对公共危机和突发事件的能力。加快建立和完善重大问题集体决策制度、专家咨询制度、决策责任制度，推进科学民主决策。坚持执政为民，树立正确的政绩观，大兴求真务实之风，深入实际调查研究，狠抓各项工作落实。建立和完善干部政绩考核体系和奖惩制度，认真落实工作责任制，把目标任务分解量化，确保责任落到实处。加强审计监督，切实落实廉政建设责任制，从源头上预防和治理腐败。积极筹建市行政服务中心，推进机关效能建设。加强电子政务、政务公开和社会保障等信息网络建设，提高办事效率和办事水平。加大对违纪违法案件的查处力度，坚决纠正损害群众利益的不正之风。加强公务员队伍建设，提高公务员素质。牢记“两个务必”，落实“五个力戒”，干净干事，说实话，办实事，鼓实劲，出实效，努力树立“为民、务实、清廉”的政府新形象。

　　各位代表！揭阳正处在抢抓机遇、加快发展的关键时期，我们肩负着光荣而又艰巨的历史重任。让我们高举邓小平理论伟大旗帜，按照“三个代表”重要思想的要求，在中共揭阳市委的正确领导下，振奋精神，迎难而上，开拓创新，扎实工作，为我市全面建设小康社会、实现跨越式发展而努力奋斗！
